# Investigation for atherosclerotic plaque rupture with thrombosis in mice based on single-cell sequencing and bioinformatics analysis

**DOI:** 10.3389/fcvm.2025.1658170

**Published:** 2025-12-03

**Authors:** Peng Nie, Fang Wan, Tianbao Yao, Yao Li, Guofeng Yan, Jun Pu, Shuxuan Jin

**Affiliations:** 1 Division of Cardiology, Renji Hospital, Shanghai Jiao Tong University School of Medicine, Shanghai, China; 2 Department of Laboratory Animal Science, Shanghai Jiao Tong University School of Medicine, Shanghai, China

**Keywords:** atherosclerosis, plaque rupture, thrombosis, mouse model, hub gene, functional and pathway analysis

## Abstract

**Introduction:**

Atherosclerotic plaque rupture with thrombus formation leads to severe cardiovascular events. We hoped to explore the hub genes providing critical roles in the process of atherosclerotic plaque rupture accompanied by thrombus formation, which might provide a new research direction for clinical therapy.

**Methods:**

The mouse model of atherosclerotic plaque rupture with thrombosis was established by the combined ligation of the left renal artery and common carotid artery, while key genes and regulatory pathways were identified using single-cell RNA sequencing and bioinformatics analysis.

**Results:**

The mice model accompanied by atherosclerotic plaque rupture with thrombus formation was successfully established. Seventeen cell subsets were identified based on scRNA-seq analysis including Fibroblasts. Downstream analysis showed 376 TDEGs were revealed to be closed associated with thrombosis-prone plaques. These TDEGs were mainly enriched in cell adhesion. A total of five hub genes including *COL5A1*, *VCAN*, *PTGS2*, *ITGAV*, and *ITGA8* were investigated. Drug-gene interaction network analysis identified several drug-gene relations, such as Aspirin-PTGS2. Fibroblasts might play a vital role in atherosclerotic plaque rupture with thrombosis.

**Discussion:**

*COL5A1*, *VCAN*, *PTGS2*, *ITGAV* and *ITGA8* might be novel biomarkers for atherosclerotic plaque rupture with thrombosis. *ITGAV* and *VCAN* might take part in the process atherosclerotic plaque rupture with thrombosis via cell adhesion function.

## 1 Introduction

Atherosclerosis is a chronic vascular disease characterized by the deposition of lipids and inflammation within arterial blood vessels, frequently leading to cardiovascular events (1). Notably, the rupture of atherosclerotic plaques, coupled with subsequent thrombus formation, stands as a primary trigger for life-threatening complications, such as acute myocardial infarction and stroke (2). Despite considerable understanding of the formation and progression of atherosclerotic plaques (3), the precise mechanisms underlying plaque rupture with thrombus formation remain unclear.

Single-cell RNA sequencing (scRNA-seq) provided a high-resolution method of cell populations at the single-cell level (4). In recent years, the development of single-cell sequencing technology has provided a crucial tool for unraveling the functions and interactions of different cell types within complex tissues (5). A previous study showed that scRNA-seq could be successfully used for the multicellular ecosystem investigation in vena caval tumor thrombus in clear cell renal cell carcinoma (6). Certain gene signatures (such as *SAT1*, *CLIC1* and *PNP*) has been revealed to be specific in human disease such as acute pancreatitis by using scRNA-seq combined with bioinformatics analysis (7). In addition, a previous scRNA-seq analysis in endometrial carcinoma proves that both cell-cell communication and pathway expression are dramatically influenced among different cell sub-clusters (8). Based on mice model, a previous scRNA-seq by Yang et al. revealed a mechanism underlying the susceptibility of the left atrial appendage to intracardiac thrombogenesis during atrial fibrillation (9). A d-flow–induced model of mouse atherosclerosis was firstly established by Nam et al. (10), thereafter, the value of animal model for plaque rupture and thrombosis has already been proved by previous study (11). In our previous study, a murine model of spontaneous plaque rupture with a high incidence of luminal thrombus has been successfully constructed (12). The model not only nicely recapitulates the pathophysiological processes of human plaque rupture but is also simple to establish, rapid to prepare, and highly efficient to generate. Thus, based on scRNA-seq technology and bioinformatics analysis, it is possible to comprehensively reveal the key cellular populations and associated molecular functions/pathways in the process of atherosclerotic plaque rupture accompanied by thrombus formation.

In the current study, a mice model of atherosclerotic plaque rupture with thrombus was constructed, followed by scRNA-seq. Then, the bioinformatics analysis was performed on the sequencing data to identify the cell subsets, hub genes and associated pathways that play a key role in the process of atherosclerotic plaque rupture accompanied by thrombus formation, and provide a new research direction for clinical therapy.

## 2 Materials and methods

### 2.1 Animal model construction and grouping

A total of 32 female ApoE-deficient (*ApoE*^−/–^) C57BL/6 mice (10 weeks old) were obtained from the Jackson Laboratory (Bar Harbor, ME). According to method in our previous study (12), all mice underwent combined partial ligation of left renal artery and left common carotid artery (LCCA) to establish the mice model of atherosclerotic plaque rupture with thrombosis. All mice were anesthetized and euthanized 8 weeks after surgery using 4% isoflurane inhalation, in accordance with the AVMA Guidelines for the Euthanasia of Animals (2020 edition). Following euthanasia, the animals were subjected to a whole-body perfusion with phosphate-buffered saline (PBS). Upon examination of the left carotid artery under a microscope, all mice exhibited the presence of atherosclerotic plaques. Notably, thrombosis was observed in 16 of these mice, while the remaining 16 exhibited significant plaque formation without thrombosis. Consequently, the mice were classified into two experimental groups based on the presence of thrombus: the thrombus group (*n* = 16) and the vulnerable plaque group (*n* = 16). For further analyses, both groups were subdivided equally into the single-cell sequencing group and the pathological section group, respectively. Carotid artery specimens from both sides (right and left) were collected from all 32 mice for downstream processing. Moreover, the specimens were then distributed into three groups for single-cell RNA sequencing. Right carotid arteries from the 16 mice were combined and served as the control group (A1 group). Left carotid artery samples from eight mice without thrombus formation but with plaques were pooled as A2 group, and left carotid artery samples from eight mice with thrombus formation were served as A3 group. In addition to sequencing, tissue sections from the left carotid arteries of eight mice with plaques but without thrombosis and other eight mice with thrombus formation were embedded for cryosectioning and further histological analysis. All surgeries were performed under the dissecting microscope. Mice were provided with a standard rodent diet and tap water *ad libitum* during the experiment.

All animal work was performed in accordance with the guidelines on animal care of Shanghai Jiao Tong University School of Medicine. The experimental protocol was approved by the Institutional Animal Care and Use Committee (IACUC) of Shanghai Jiao Tong University School of Medicine (Approved number: JUMC2023-059-B).

### 2.2 Single-cell RNA sequencing

Using a dissecting microscope, the isolated vascular tissue was carefully chopped and incubated in a solution containing dissociation enzyme (1 mg/ml collagenase type II, 0.02 mg/ml Deoxyribonuclease I) for 1 h at 37°C. Thereafter, three rounds of centrifugation-washing (500×g for 5 min each) with ice-cold PBS containing 0.04% BSA were performed to remove cell-free RNA released from disrupted cells or residual dissociation reagents. Then, the cell suspension was filtered through a 40 µm sterile cell strainer (JETBIOFIL, Cat#css010040), which not only removed cell clumps but also reduced free-floating RNA fragments. To prepare a single-cell suspension, 0.12% trypsin solution was used to resuspend cells. Based on AOPI Dual-ﬂuoresces counting, cell activity above 90% (primary cell activity above 70%) and concentration between 300 and 600/μl cells were used for sequencing. The single-cell RNA sequencing process was provided by Renji Hospital Affiliated to Shanghai Jiao Tong University School of Medicine. Briefly, the prepared single cell suspension is combined with a mixture of gel beads containing barcode information and enzymes, so as to form GEMs (Gel Bead in EMulsions) containing glue beads (with prefabricated 10× primers), single cells and Master Mix. Single cells were resuspended in PBS with 0.04% BSA and added to each channel. The captured cells were lysed, and the released RNA was barcoded through reverse transcription in individual GEMs. Barcoded cDNA was ampliﬁed, and the quality was controlled using Agilent 4200TapeStation System, followed by the scRNA-seq libraries preparation. Then, sequencing was performed on an Illumina Novaseq 6000 sequencer with a pair-end 150 bp (PE150) reading strategy. Finally, the Sequenced Reads were obtained after the sequencing was completed.

### 2.3 Single-cell data preprocessing

The data preprocessing was performed based on sequenced reads obtained above. Briefly, the raw data of single-cell sequencing was obtained in fastq format, followed by quality assessment and alignment to a reference genome. Subsequently, data quality control was conducted to filter out high-quality cells with gene detection counts between 200 and 7,000, as well as a mitochondrial gene ratio below 15%. DecontX algorithm via the celda R package was applied to distinguish between true intracellular gene expression and ambient RNA contamination by leveraging the expression patterns of marker genes and the distribution of gene counts across cells with the contamination probability threshold to 0.05 (13). Then, the data was integrated using the seurat package in R (version: 3.1.2) with Canonical Correlation Analysis (CCA) to correct the batch effects. Next, the data was standardized using global scaling normalization with the LogNormalize method, followed by the linear dimension reduction via Principal Component Analysis (PCA). Finally, the K-Nearest Neighbors (KNN) algorithm from the Seurat package in R was used to cluster cells, followed by Uniform Manifold Approximation and Projection (UMAP) non-linear dimension reduction.

### 2.4 Cell clustering, development, and communication analysis

According to genes associated with mouse cell clustering reported in previous literature, the scMCA and CellMarker2.0 software were used to acquire markers associated with mouse cell clustering, followed by annotation. Subsequently, Pearson correlation coefficients were computed between cell subgroups, and a heatmap depicting the correlation was generated to conduct subgroup correlation analysis. To analyze fibroblast cell development, we employed Monocle2 software to perform pseudotime trajectory analysis. Based on prior clustering analyses, three distinct fibroblast subtypes were identified: Fibroblasts, *Pi16+* Fibroblasts, and *lsg* *+* Fibroblasts. Using Monocle's differential analysis, we selected pseudotime-related genes to capture key expression patterns critical to cell differentiation. By learning the sequence of gene expression changes that each cell undergoes, individual cells were ordered according to their pseudotime values. This allowed us to simulate the dynamic developmental process, aligning each cell along its respective trajectory. Cells were subsequently categorized into multiple differentiation states based on gene expression profiles, and a visual lineage tree was constructed to predict cellular differentiation and developmental pathways. Finally, based on human homologous genes of mouse origin, the iTALK package in R software was employed to analyze receptor-ligand interaction networks among various states and within each state.

### 2.5 DEGs investigation

Based on different cell cluster, the cluster biomarkers (DEGs) between groups were revealed by using FindMarkers of seurat package in R software. The min.pct = 0.25 and logfc.threshold = 0.25 were used as the cut-off values for cluster biomarkers revealing. Genes with *P* < 0.05 and | log Fold Change (FC) | > 1 in A1 vs. A2 and A1 vs. A3 were investigated as DEGs in current study. The results of DEGs were visualized by volcano plot using ggplot software (Version: 3.0.0) (14). Finally, the VENN plot analysis was further performed on these DEGs to explore thrombus-related DEGs (TDEGs) using jveen software (15).

### 2.6 Enrichment and PPI network analysis based on TDEGs

GO function and KEGG pathway analyses were performed based on the TDEG using clusterProfiler package (version: 3.16.0) (16) of R. The GO functions including biological process (BP), cellular components (CC), and molecular function (MF). *P* value < 0.05 was considered as the thresholds for current enrichment analysis. Moreover, according to STING database (version: 11.0) (17), the protein interaction information was extracted, and PPI pairs (median confidence = 0.7) among TDEGs in this study were predicted. Finally, the PPI network was constructed by Cytoscape (version: 3.6.1) software (18).

### 2.7 The prediction of miRNA for hub genes

The common TDEGs in at least 2 cell clusters were considered as hub genes in current study. Then, the miRNAs that target with hub genes were predicted using TargetScan and miRDB in miRWalk software (version: 3.0) (19), followed by the miRNA-hub gene interaction network construction. Finally, the results were visualized by Cytoscape software.

### 2.8 Transcription factors (TFs)-gene interaction network investigation

The upstream TFs of hub genes were predicted using iRegulon in Cytoscape software. The *P* < 0.05 was selected as the thresholds for TFs investigation. Finally, the TFs-hub gene network was visualized by Cytoscape software.

### 2.9 Drug-gene interaction prediction

The drugs targeted by homologous human genes of hub genes were screened using Drug-Gene Interaction database (DGIdb, version: 4.0) (20). Based on the drug-target gene relations, the drug-target gene interaction network was constructed using Cytoscape software (version: 3.9.2).

### 2.10 Sample collection and qPCR

Human carotid artery tissue samples were collected from patients who underwent carotid endarterectomy or autopsy at the Division of Cardiology, Renji Hospital. The study protocol was approved by the Ethics Committee of Renji Hospital, Shanghai Jiao Tong University School of Medicine. Carotid artery tissues from patients without atherosclerotic lesions (*n* = 8), with stable atherosclerotic plaques (*n* = 10), and with ruptured atherosclerotic plaques and intraluminal thrombosis (*n* = 12) were collected.

qPCR was performed using the SYBR Premix Ex Taq II Kit (TaKaRa Bio, Otsu, Japan) on a StepOnePlus Real-Time PCR System (Thermo Fisher Scientific, Waltham, MA, USA) to detect the relative expression levels of the target genes (COL5A1, VCAN, PTGS2, ITGAV, and ITGA8) and the internal reference gene (GAPDH). The relative expression level of each target gene was calculated using the 2^−ΔΔCt^ method.

## 3 Results

### 3.1 Histological observation among different groups

**Figure 1:**
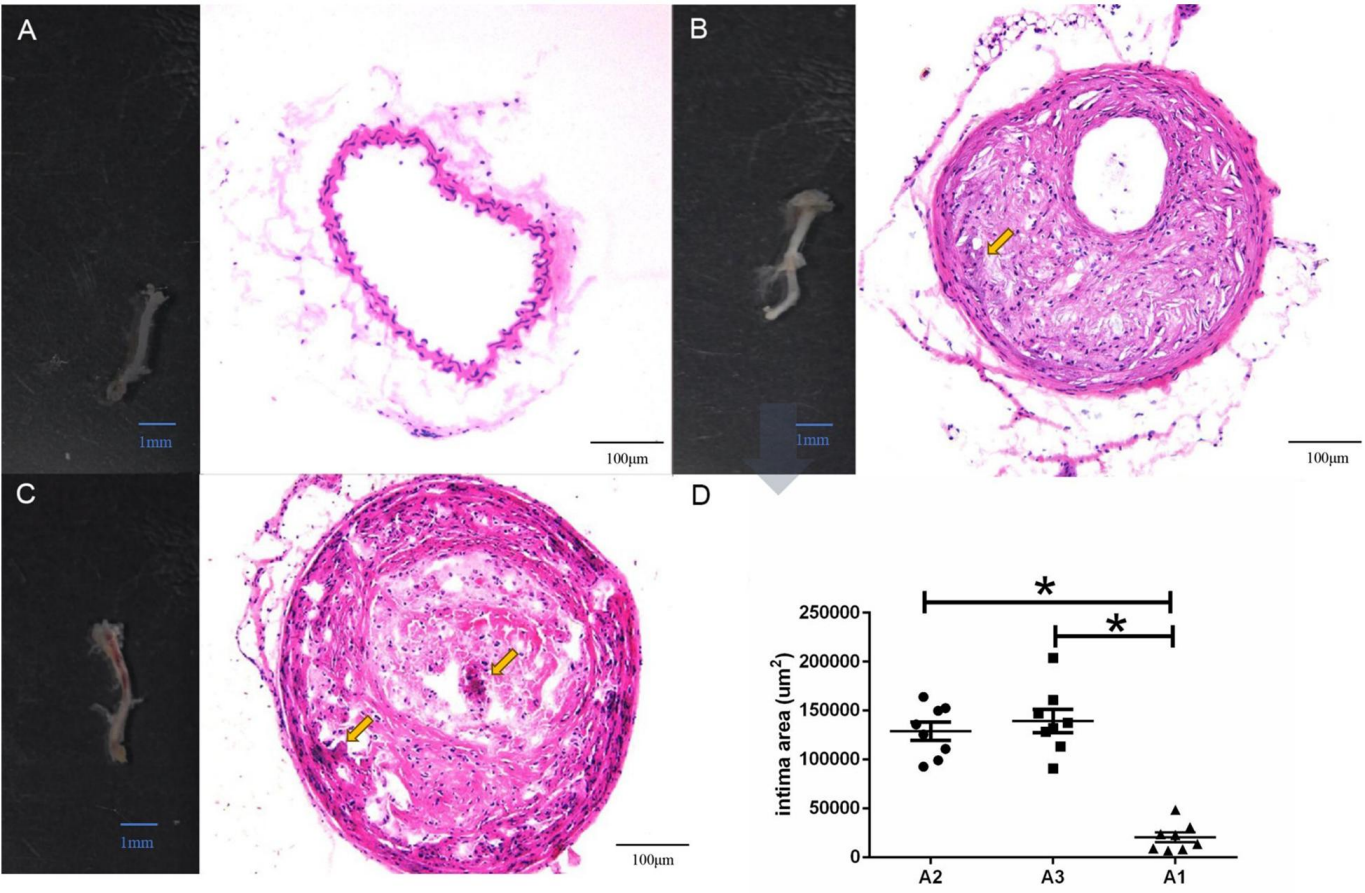
Histological observation among different groups based on established mice model using partial ligation of left renal artery and left common carotid artery (LCCA). **(A)**, control group (A1 group, *n* = 16): representative image of the right carotid artery (scale bar = 1 mm) and its corresponding hematoxylin-eosin (HE) staining (scale bar = 100 μm). No atherosclerotic plaques or thrombi were observed in the arterial wall. **(B)**, plaque-only group (A2 group, *n* = 8): representative image of the left carotid artery (scale bar = 1 mm) and its HE staining (scale bar = 100 μm). Atherosclerotic plaques (pointed by the yellow arrow) were visible in the arterial intima, with no thrombus formation. In the figures, 1–24 represent fibroblasts, Pi16+ fibroblasts, Isg + fibroblasts, SMCs, modulated SMCs, endothelial cells, cytokine-simulated endothelial cells, lymphatic endothelial cells, Trem2+ macrophages, inflammatory macrophages, T cells, B cells, pericytes, neurons, adipocytes, cycling cells, Fabp4+ endothelial cells, fibroblasts, SMCs, Trem2+ macrophages, T cells, Isg + fibroblasts, cytokine-simulated endothelial cells, cycling cells, respectively. **(C)** Thrombus group (A3 group, *n* = 8): representative image of the left carotid artery (scale bar = 1 mm) and its HE staining (scale bar = 100 μm). Both atherosclerotic plaques and intraluminal thrombi were observed (both pointed by the yellow arrows). **(D)** Quantification of the intimal surface area of atherosclerotic lesions: the *x*-axis indicates the three groups (A1, A2, A3), and the *y*-axis represents the intimal area (unit: μm^2^). Data are presented as mean ± standard deviation; *P < 0.05 compared with the A1 group (control group).

The tissue sections of A1, A2 and A3 group used for histological analysis. The result showed that when compared with A1 group (Figure 1A), the plaque and thrombus could be observed in A2 group (Figure 1B) and A3 group (Figure 1C), respectively. The comparative analysis of intima area showed that when compared with A1 group, there was a significant increase of intima area in both A2 group and A3 group (all *P* < 0.05) (Figure 1D). All results indicated that the mice model established by using LCCA in current study could successfully induce atherosclerotic lesions with plaque disruption associated with lumen thrombosis.

### 3.2 Quality control (QC) on sequencing data

After QC on A1 group, A2 group and A3 group, the high-quality single cells were obtained and kept for downstream analysis. Additionally, we applied Scrublet to identify potential doublets. Overall, the QC process effectively removed low-quality cells and genes, improving the overall quality of current data.

### 3.3 Cell clustering, development and communication investigation

**Figure 2:**
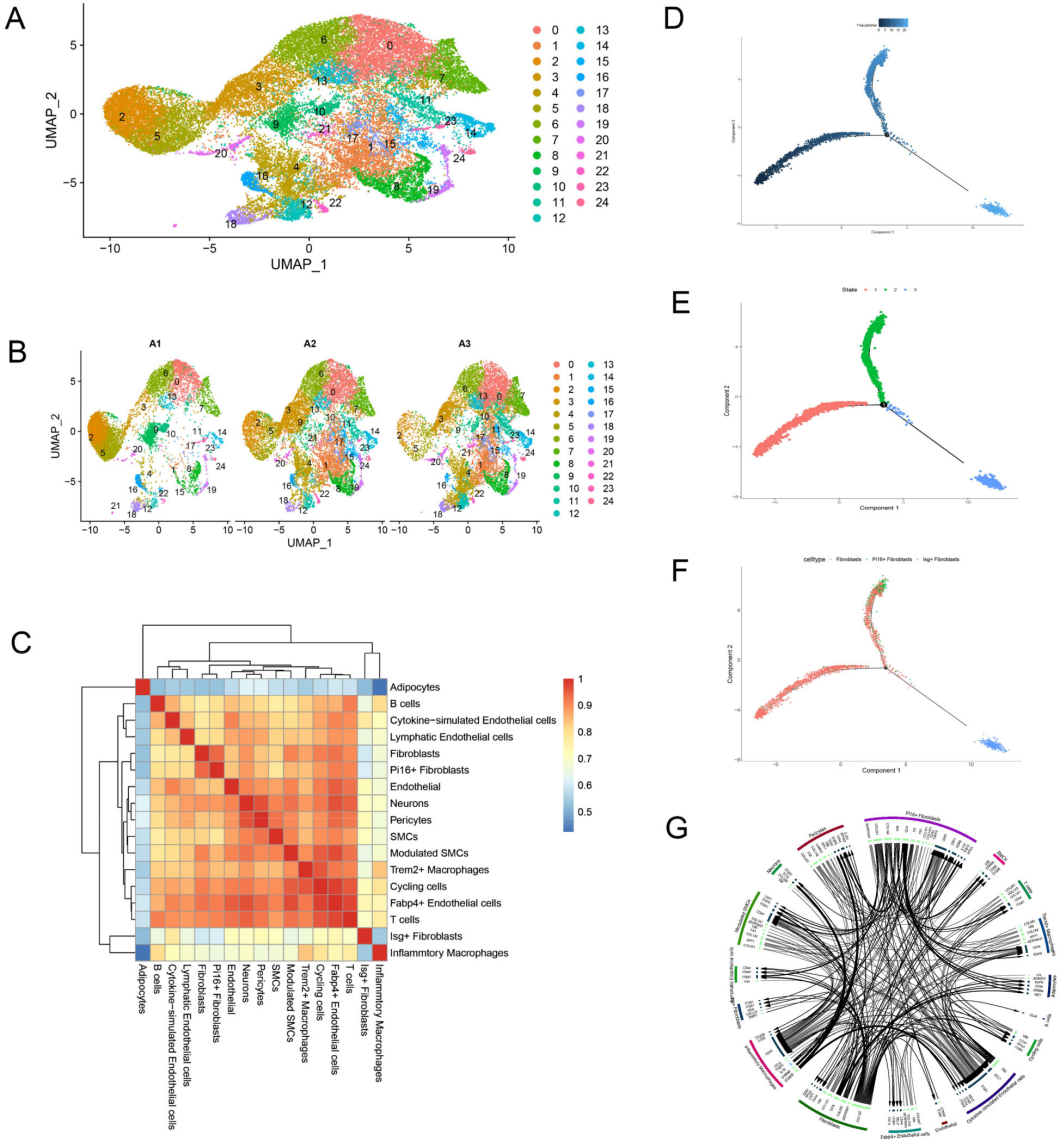
Results of cell clustering, developmental trajectory, and cell-cell communication analysis based on single-cell RNA sequencing (scRNA-Seq). **(A)** Uniform manifold approximation and projection (UMAP) plot showing the distribution of 17 distinct cell subtypes. Each color represents a unique cell type, and the *x*-axis (UMAP_1) and *y*-axis (UMAP_2) represent the two-dimensional dimensionality reduction components. **(B)** Distribution of cell subtypes across the three groups. UMAP plot overlaid with group labels (A1: control, A2: plaque-only, A3: thrombus). The color coding for cell subtypes is consistent with **(A)**, showing how the composition of cell types varies among groups. **(C)** Correlation heatmap among cell subtypes. The color intensity represents the Pearson correlation coefficient between different cell types (darker colors indicate stronger correlations). This heatmap reflects the co-expression patterns and potential functional associations between cell subtypes. **(D)** Pseudotime trajectory of fibroblasts: the result showed the progression of cells from the root through multiple bifurcation points (marked as 1) along the pseudotime axis, with earlier stages on the left and later stages on the right. **(E)** Cellular state distribution along the pseudotime trajectory: the result showed the different differentiation states with bifurcation points marked as 1, where each state was represented between bifurcations and vertices. **(F)** Cell type distribution along the pseudotime trajectory: fibroblasts (red), *Pi16+* fibroblasts (green), and *lsg* *+* fibroblasts (blue), with bifurcation points marked as 1. **(G)** The receptor-ligand communication relationship among states and in each state.

Top 10 principal components (PCs) were revealed by using Elbow-plot analysis (Supplementary Figure 1A). Then, these 10 PCs were used for cell clustering based on UMAP nonlinear dimensionality reduction (Supplementary Figure 1B). The result showed that there were totally 25 clusters revealed by UMAP analysis. The detail information of clustering in each group was showed in Supplementary Figure 1C. Then, the obtained cells were classified based on the marker gene of mice. The results showed that a totally 17 cell subsets were annotated in current study (Figure 2A). In the carotid artery tissues of control (A1), plaque-only (A2), and thrombus (A3) groups. These subsets include not only well-characterized cell types in atherosclerosis, such as endothelial cells, SMCs, Trem2 + macrophages, T cells, but also three fibroblast subtypes, such as Fibroblasts, Pi16+ Fibroblasts, Isg + Fibroblasts, and specialized populations like “Cytokine-simulated Endothelial cells” and “Modulated SMCs.” Furthermore, the detail information for cell types in all three groups (A1, A2 and A3) revealed that pro-inflammatory populations, such as inflammatory macrophages and Isg + Fibroblasts, are nearly absent in A1 but significantly expanded in A2 and A3, while fibroblasts consistently represented the largest cell fraction across all groups, suggesting their central role in both plaque formation and thrombus progression (Figure 2B). In addition, the result of correlation analysis among cell types were showed in Figure 2C.

Fibroblasts are critical for extracellular matrix (ECM) synthesis and tissue repair, but their developmental trajectory and subtype-specific roles in atherosclerotic thrombosis remain unclear. The pseudotime analysis revealed distinct developmental trajectories among the fibroblast cell types. Cells were arranged according to their pseudotime along the inferred trajectories, with early-stage cells situated near the root (Figure 2D). This analysis enabled the identification of several bifurcation points, indicating critical decision points in cell fate determination. The fibroblast cell types displayed differential expression patterns that corresponded to unique developmental states (Figure 2E). The results suggested a clear developmental hierarchy, with distinct fibroblast subtypes, Fibroblasts, *Pi16+* Fibroblasts, and *lsg* *+* Fibroblasts, distributed along the pseudotime trajectory (Figure 2F). In A2 (plaque-only), most fibroblasts are in the early/intermediate states (Fibroblasts, Pi16+ Fibroblasts), which express high levels of ECM-related genes consistent with plaque structural maintenance. In A3 (thrombus), fibroblasts shift toward the late Isg + Fibroblast state, which is enriched in interferon-stimulated genes and pro-inflammatory cytokines, suggesting a switch from “repair-focused” to “inflammation-amplifying” functions during thrombus formation.

Finally, the receptor-ligand communication relationship among states and in each state were showed in Figure 2G. For example, Fibroblasts/Pi16+ Fibroblasts in A2 secrete ligands like TGF-β1, which signals to SMCs via the TGFBR1/2 receptor to promote ECM synthesis. In A3, Isg + Fibroblasts and Inflammatory Macrophages secrete CXCL10 and CCL2, which bind to CXCR3/CCR2 on T cells and endothelial cells, recruiting immune cells and disrupting endothelial barrier function to facilitate thrombus formation.

### 3.4 DEGs investigation and enrichment analysis

**Figure 3:**
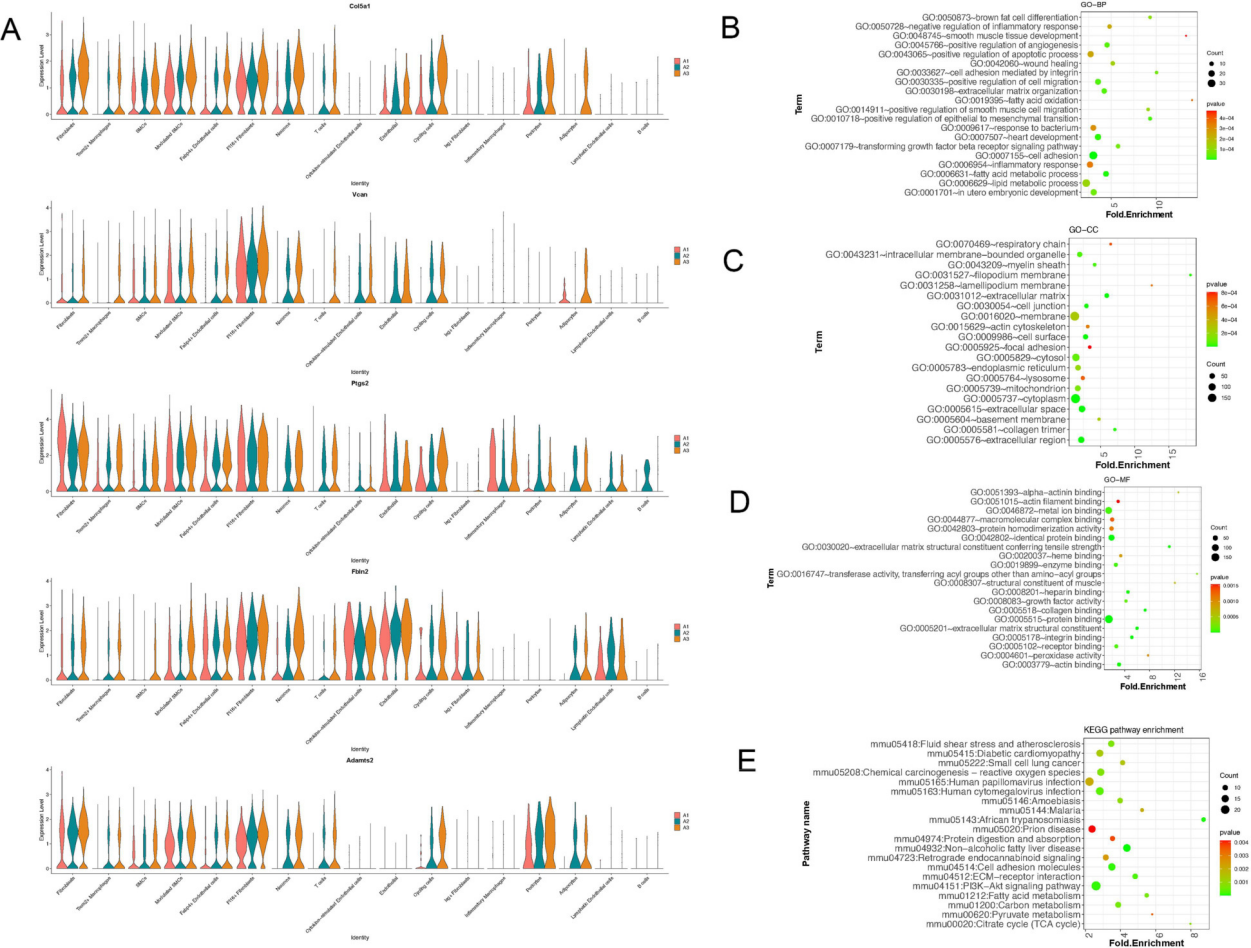
Identification of differentially expressed genes (DEGs) and their functional enrichment analysis. **(A)** Expression levels of five representative hub genes across the three groups: bar plot showing the relative expression of COL5A1, VCAN, PTGS2, ITGAV, and ITGA8 in A1 (control), A2 (plaque-only), and A3 (thrombus) groups. The *x*-axis indicates the gene names, and the *y*-axis represents the normalized expression level (log2-transformed). Data are presented as mean ± standard deviation. **(B–D)**, GO functional enrichment analysis of thrombus-related DEGs (TDEGs): bubble plots showing the top enriched GO terms in three categories, including biological process (BP), cellular component (CC), and molecular function (MF). The *x*-axis represents “Fold Enrichment”, the *y*-axis represents the GO term name, the size of the bubble indicates the number of TDEGs enriched in the term (larger bubbles = more genes), and the color intensity indicates the statistical significance. **(E)** Kyoto encyclopedia of genes and genomes (KEGG) pathways of TDEGs. The *x*-axis represents “Fold Enrichment”, the *y*-axis represents the pathway name, and the bubble size/color follows the same rules as **(B–D)** (*P* < 0.05).

The DEGs in each cell subset, compared to other categories, were identified as biological marker genes for corresponding cell subset. The results showed that 812 DEGs were revealed in the A2 vs. A1 comparison, while 1,004 DEGs were identified in the A3 vs. A1 comparison. Meanwhile, a total of 1,004 DEGs were explored between A3 group and A1 group. Due to the large number of genes involved, we presented the results for five representative genes, including *COL5A1*, *VCAN*, *PTGS2*, *ITGAV*, and *ITGA8* (Figure 3A). Based on these DEGs, the VENN plot analysis further revealed 376 TDEGs (Supplementary Figure 2). These TDEGs were mainly assembled in GO functions including cell adhesion (BP, GO: 0007155, Figure 3B), membrane (CC, GO: 0016020, Figure 3C) and protein binding (MF, GO: 0005515, Figure 3D). In addition, these TDEGs were mainly enriched in KEGG pathways like Akt signaling pathway (mmu04151, Figure 3E).

### 3.5 PPI network analysis

**Figure 4:**
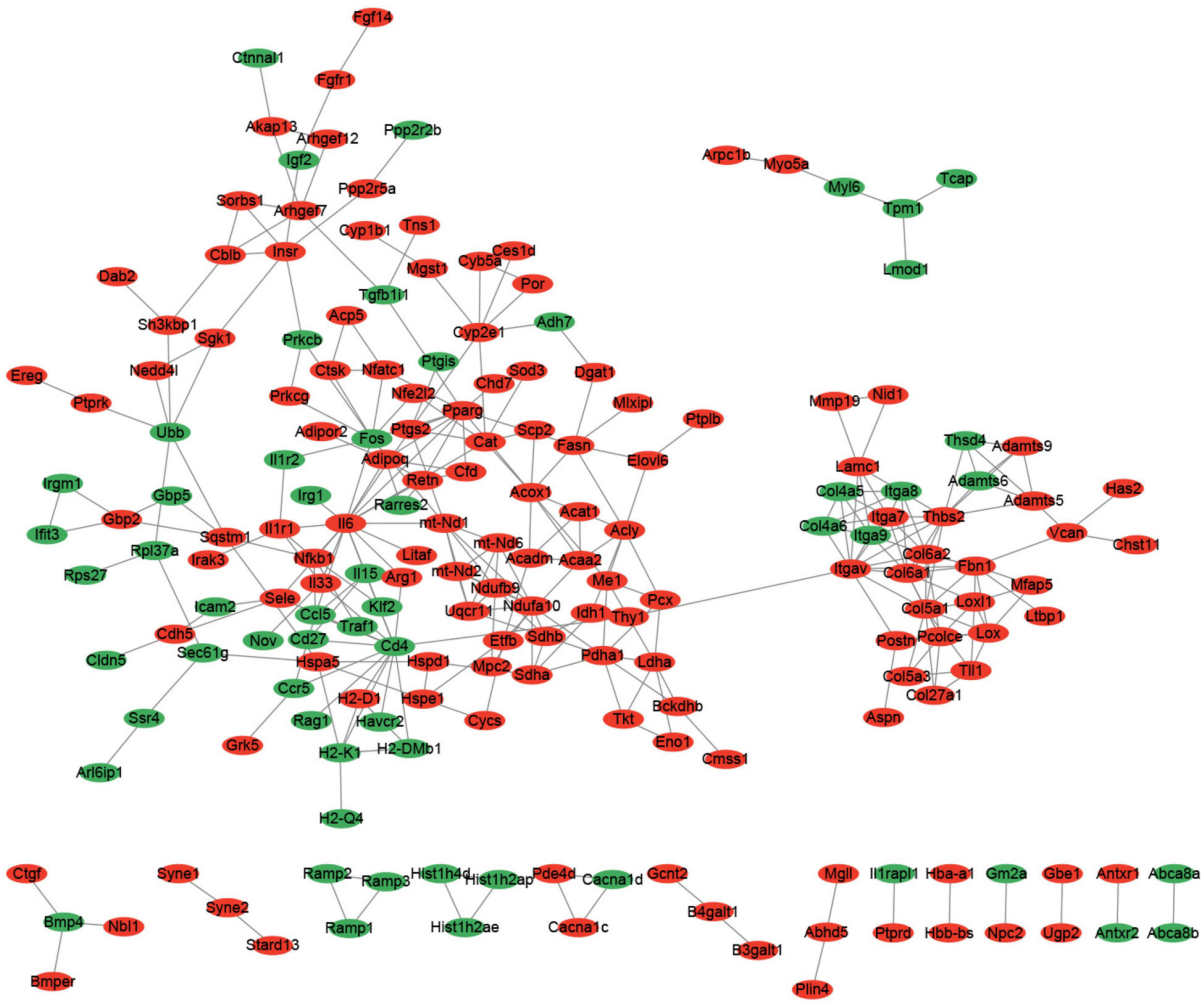
Protein-protein interaction (PPI) network constructed by TDEGs. The red node represented up-regulated gene. The green node represented down-regulated gene. The line between two nodes represented interaction.

A PPI network was constructed based on TDEGs. The result showed that there were 190 nodes and 319 interactions in current PPI network (Figure 4). According to the degree of these nodes, the Top 20 nodes including *IL6*, *CD4* and *ITGAV* were selected as hub nodes (Supplementary Table 1 in Supplementary Information).

### 3.6 The miRNA-mRNA interaction network analysis

**Figure 5:**
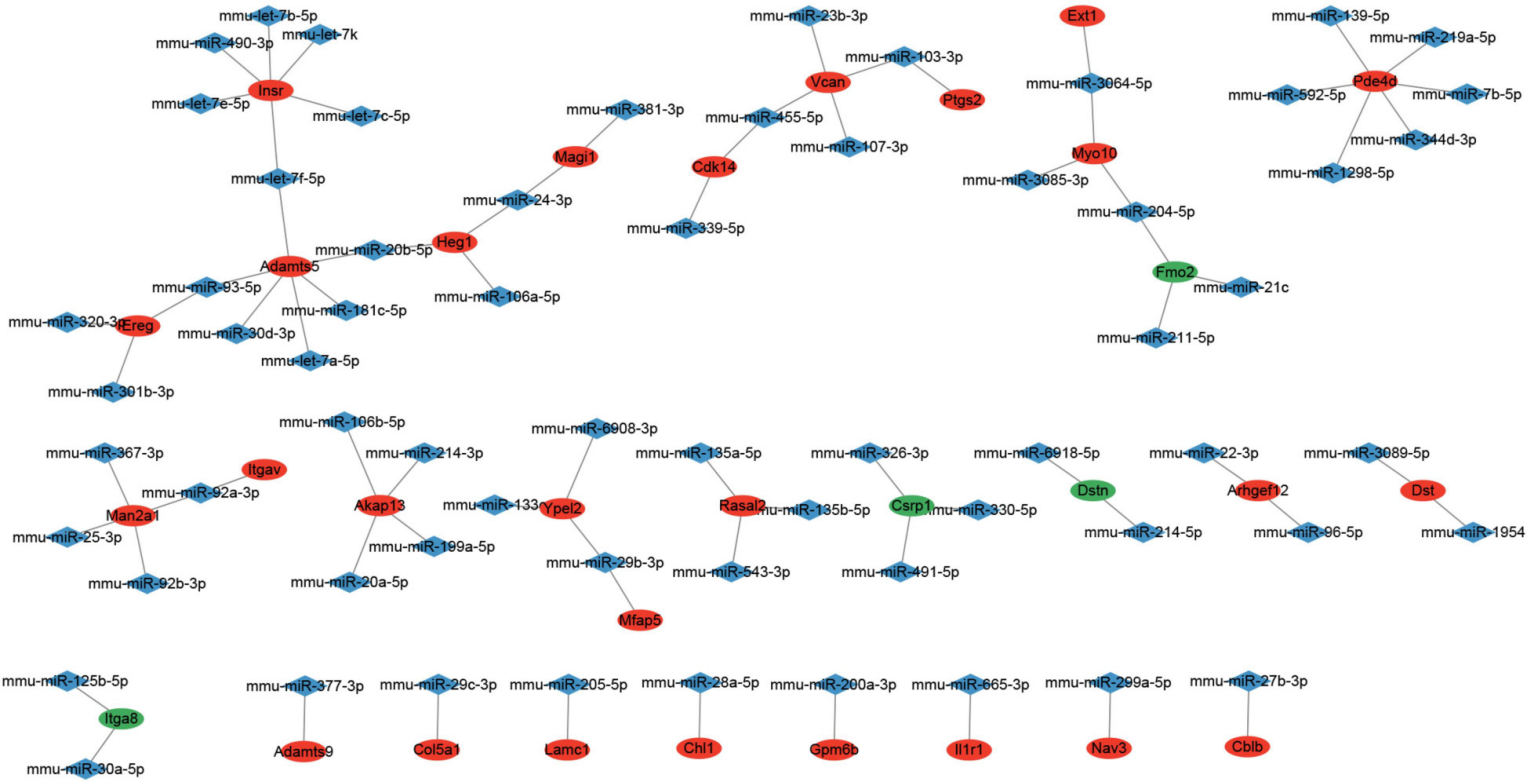
MiRNA-mRNA interaction network of hub genes. The red node represented up-regulated mRNA. The green node represented down-regulated mRNA. The blue node represented miRNA. The line between two nodes represented interaction.

The common TDEGs in at least 2 cell clusters were enrolled as hub genes, followed by totally 65 miRNAs that target with hub genes predicted. Then, a miRNA-mRNA interaction network was constructed with 4 down-regulated mRNAs, 27 up-regulated mRNAs and 65 miRNAs (Figure 5).

### 3.7 Transcription factors (TFs)-gene interaction network analysis

**Figure 6:**
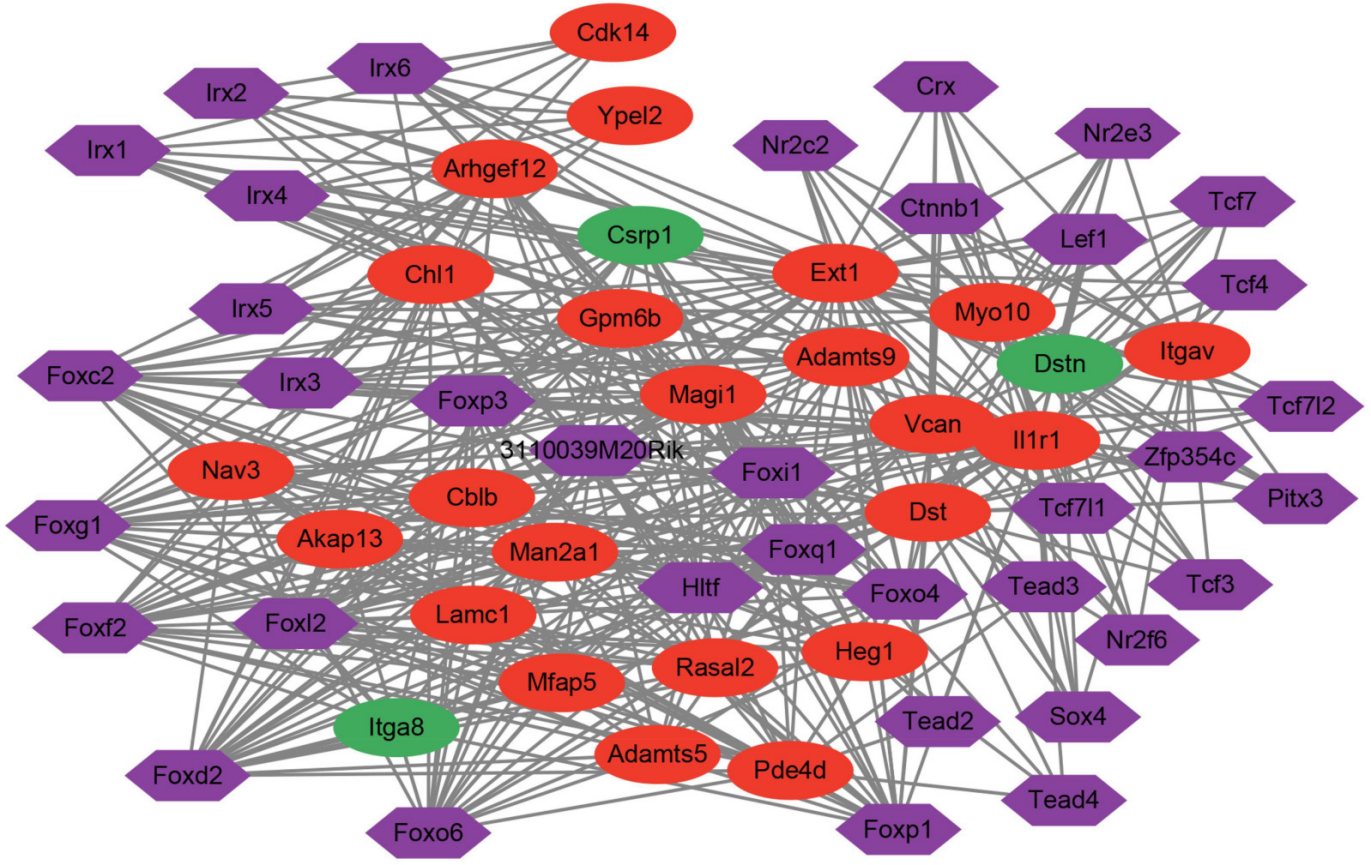
Transcription factors (TFs)-gene interaction network of hub genes. The red node represented up-regulated gene. The green node represented down-regulated gene. The purple node represented TF. The line between two nodes represented interaction.

With *P* < 0.05, a total of 36 TFs of hub genes were predicted. Then, a TFs-hub gene network was visualized by Cytoscape software (Figure 6). The result showed that there were 36 TFs, 23 up-regulated genes and 3 down-regulated genes in current network.

### 3.8 Drug-gene interaction network analysis

**Figure 7:**
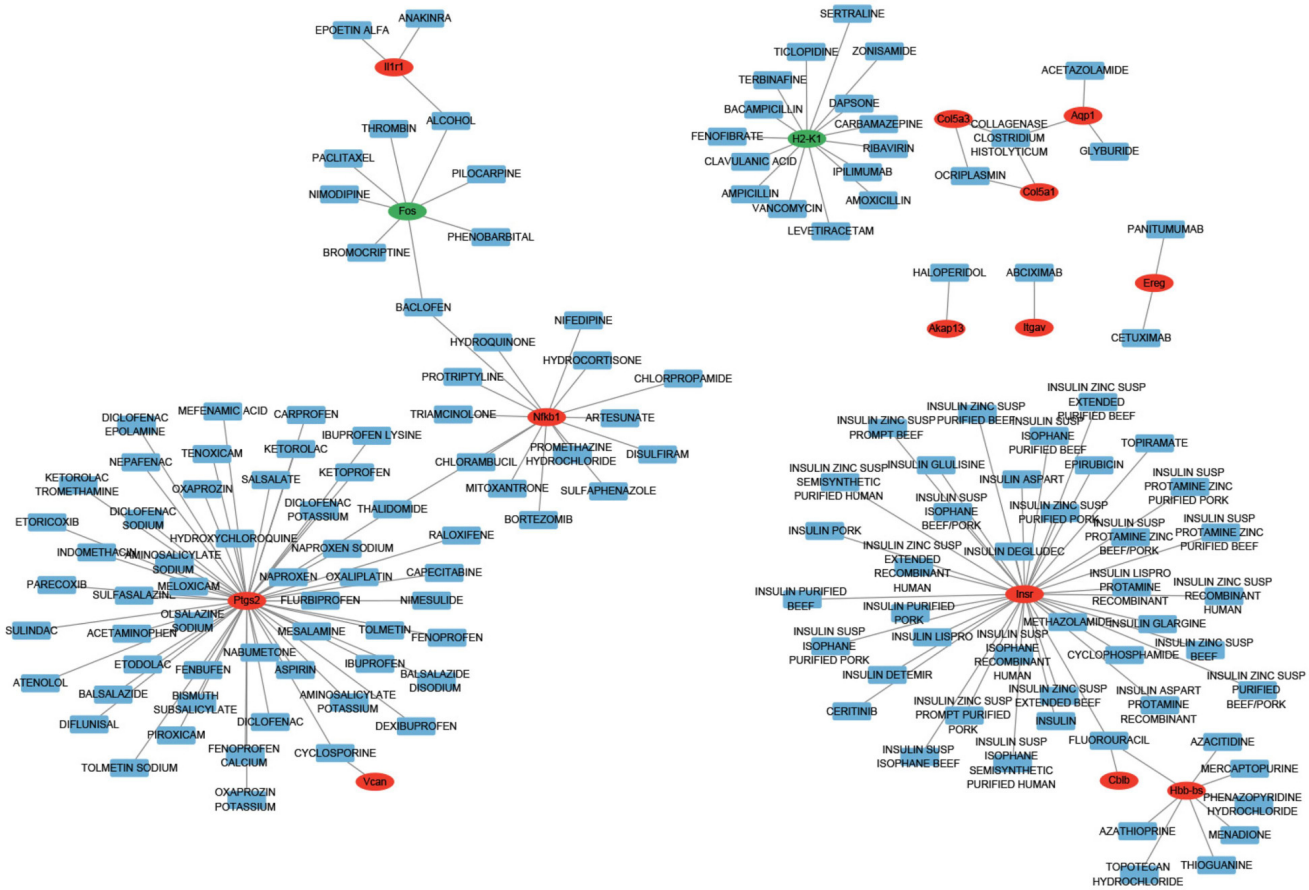
Drug-gene interaction network of hub genes. The red node represented up-regulated gene. The green node represented down-regulated gene. The blue node represented drug for thrombus.

A total 143 drugs targeted by homologous human genes of hub genes were screened using DGIdb. Then, a drug-gene interaction network was constructed with several drug-gene relations such as Aspirin-PTGS2 (Figure 7). The result showed that there were 143 drugs, 13 up-regulated genes and 2 down-regulated genes in current network.

### 3.9 The levels of five hub genes in clinical subjects

**Figure 8:**
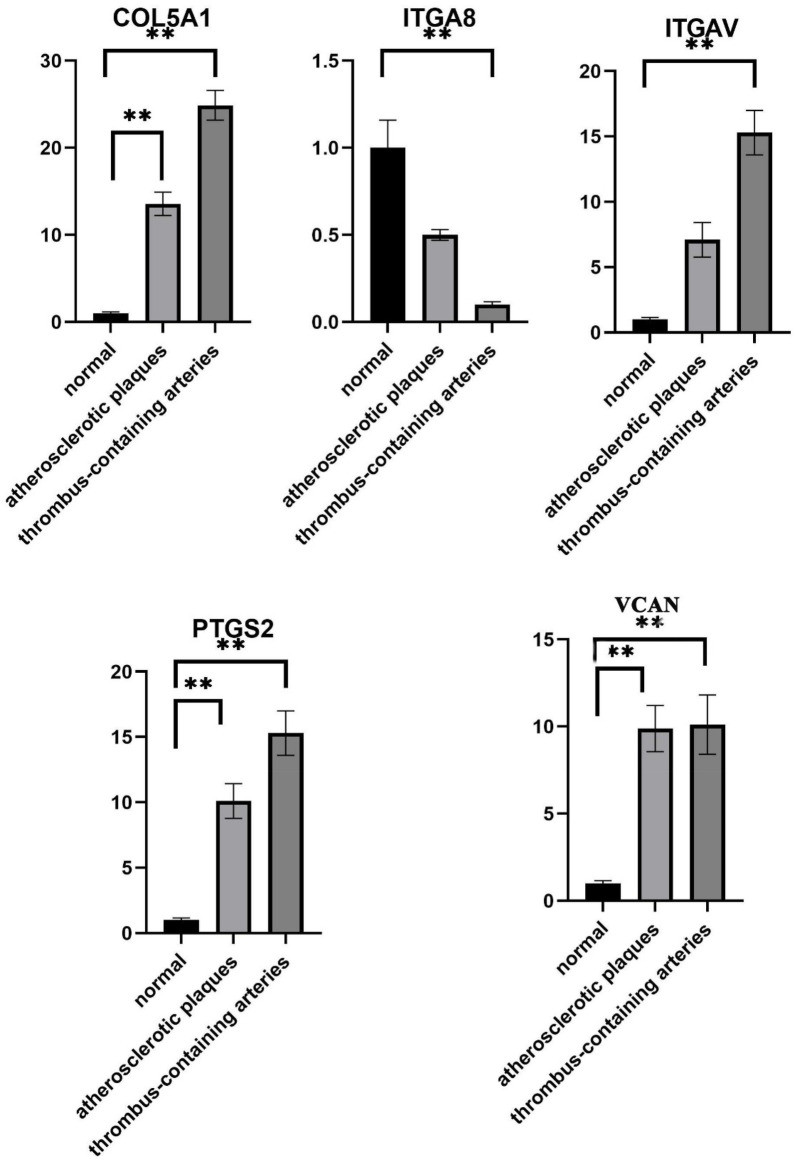
Relative expression levels of COL5A1, ITGA8, ITGAV, PTGS2, and VCAN in different clinical groups detected by qPCR.

As shown in Figure 8, the levels of five hub genes in clinical subjects were investigated. Compared with the normal group, the expression of all five genes was significantly upregulated in the plaque group, and the upregulation was more pronounced in the plaque rupture with thrombosis group. Among them, COL5A1, VCAN, PTGS2, and ITGAV showed extremely significant increases (*P* < 0.01); ITGA8 was significantly decreased in the plaque group and thrombosis group (*P* < 0.01). These findings further confirm that these genes are potential clinical biomarkers for this disease.

## 4 Discussion

The unstable atherosclerotic plaque rupture commonly leads to acute cardiovascular disease in clinical. If the mechanism of thrombosis-prone plaques could be detected and thrombosis averted, atherosclerosis would be a much more benign disease. In this study, we established a murine model of atherosclerotic plaque rupture with thrombosis, and performed single-cell sequencing on the model tissues. Based on the sequencing results, 17 cell types including fibroblasts were identified. Through bioinformatics analysis, a total of 376 TDEGs including *COL5A1*, *VCAN*, *PTGS2*, *ITGAV*, and *ITGA8* were revealed to be closed associated with thrombosis-prone plaques. These thrombosis-related DEGs were mainly enriched in pathways such as cell adhesion. Drug-gene interaction network analysis identified several drug-gene relations, such as Aspirin-PTGS2.

The rupture of atherosclerotic plaques and subsequent thrombus formation are critical steps leading to cardiovascular events, and in this process, fibroblasts may play crucial roles (21). Fibroblasts are the primary cell type of connective tissue and the main synthesizers of collagen. Within atherosclerotic plaques, they may influence plaque stability by synthesizing extracellular matrix components such as collagen (22). Upon plaque rupture, fibroblasts influence the production of new collagen for the damaged area reconstruction (23). Previous studies suggest that during plaque rupture, fibroblasts participate in inflammatory responses and repair processes, leading to thrombus formation (24). Hence, fibroblasts can produce inflammatory mediators and cytokines to modulate the extent and duration of inflammation. Following plaque rupture, fibroblasts may release pro-inflammatory mediators such as cytokines and chemokines, exacerbating the inflammatory response and further damaging the vessel wall (21). It has been proved that fibroblasts can promote thrombus formation by interacting with platelets and coagulation factors, thereby increasing the risk of vascular occlusion (25). In a previous animal experiment, Shirai et al. indicated that the cancer-associated fibroblasts could aggravate venous thrombosis via influence the platelet aggregation (26). Importantly, a previous single cell and spatial sequencing study shows that, as the major amount of cell subset, SFRP2 + fibroblasts in psoriasis contribute to amplification of the immune network through transition to a pro-inflammatory state via communicating with hub genes including CCL13, CCL19 and CXCL12 (27). By investigating protumor activities of cancer-associated fibroblasts, a previous single-cell RNA sequencing analysis proved that the pro-invasive cancer-associated fibroblast subgroup was associated with poor clinical outcomes in patients with gastric cancer (28). In this study, we found that fibroblast was one of cell types involved in the process of plaque rupture accompanied by thrombus formation in atherosclerosis. Additionally, regardless of the grouping (A1, A2 and A3), fibroblasts consistently represented the largest proportion of cell types. Thus, we speculated that fibroblasts might play a vital role in atherosclerotic plaque rupture with thrombosis. Investigating further into the specific role of fibroblasts in this disease will contribute to a better understanding of the pathogenesis and offer novel strategies for future therapies.

It has been proved that the expression of certain genes contributes to the tumor growth and increased risk of venous thrombosis in mice (29). *COL5A1*, a gene encodes collagen V-α chains, is crucial for the formation and stability of collagen fibers. A previous study showed that the variation of *COL5A1* was related to atherosclerosis, which may increase the risk of plaque formation and rupture by affecting the structure and stability of arterial wall (30). Richer et al. showed that *COL5A1* genetic variant was correlated to fibromuscular dysplasia and dysplasia-associated arterial disease, which further indicated the important role of *COL5A1* in the formation of thrombus after rupture of atherosclerotic plaque (31). *PTGS2* is an important inflammatory mediator involved in the synthesis of prostaglandins. Zhou et al. indicated that PTGS2 is the hub gene in human coronary artery atherosclerosis, which can be used as biomarkers for the severity of atherosclerosis (32). A Multi-omics and network pharmacology study proved that PTGS2 was a common gene in thrombosis induced by ischemic stroke (33). *ITGAV* and *ITGA3* are members of the integrin family involved in the adhesion between cells. Previous studies proved that *ITGAV* participated in various progression of human diseases such as the inflammation of rheumatoid arthritis and the development of liver fibrosis (34). Moreover, the high expression of *ITGAV* was closed associated with the lower overall survival of human cancer like head and neck squamous cell carcinoma, which was considered as a valuable biomarker in clinical (35). It has been proved that *ITGA3* is associated with immune process, and serves as a prognostic biomarker in human disease (36). Importantly, the biological function of *ITGAV* is commonly realized by certain biological function such as cell adhesion. Frank et al. indicated that *ITGAV* bind osteopontin was benefit for trophoblast via participating in cell adhesion (37). *VCAN* encodes brain hormone like glycoprotein, which is an important matrix protein involved in the construction of extracellular matrix and cell adhesion. *VCAN* is described to be associated with various diseases such as thrombus (38). It has been found to be associated with atherosclerosis, and its expression in plaques is associated with plaque instability and risk of rupture (39). A previous study showed that *VCAN* combined with miRNA-30a-5p could arrest tumor metastasis via cell adhesion in lung adenocarcinoma (40). In the current study, *COL5A1*, *VCAN*, *PTGS2*, *ITGAV* and *ITGA8* were five hub genes revealed to be closed associated with thrombosis-prone plaques. Meanwhile, the enrichment analysis showed that ITGAV and *VCAN* were two hub genes significantly assembled in cell adhesion function. Thus, we speculated that *COL5A1*, *VCAN*, *PTGS2*, *ITGAV* and *ITGA8* might be novel biomarkers for atherosclerotic plaque rupture with thrombosis. Moreover, *ITGAV* and VCAN might be involved in the process atherosclerotic plaque rupture with thrombosis via cell adhesion function. All these results shed light on new ideas and potential targets for the prevention and treatment of atherosclerotic plaque rupture with thrombosis.

Beyond fibroblasts, our identified key genes also imply potential roles of other cell types in atherosclerotic plaque rupture and thrombosis, particularly vascular smooth muscle cells (VSMCs). VSMCs undergo phenotypic modulation during atherosclerosis, shifting from a contractile to a synthetic state, and this process is closely associated with vascular remodeling and plaque stability. A recent study has revealed that the LXRα/UHRF1/miR-26b-3p signaling axis plays a pivotal role in regulating the phenotypic switching of VSMCs. Dysfunction of this axis may lead to extracellular matrix degradation and disruption of vascular wall stability, which is closely associated with the formation and rupture of atherosclerotic plaques (41). Notably, ITGAV, one of our prioritized genes, has been reported to be upregulated in synthetic VSMCs, where it mediates cell adhesion, migration, and extracellular matrix remodeling (42). In our scRNA-seq data, low but detectable ITGAV expression was also observed in a subset of VSMCs adjacent to fibroblast clusters, suggesting a possible cooperative role between these two cell types in regulating plaque microenvironment stability.

Aspirin is a common effective antiplatelet drug used for the prevention of recurrent thrombotic or ischemic events (43). It exerts strong antithrombotic activity in blood vessels with thrombosis by inhibiting anti-inflammatory cytokines and platelet activation (44). In animal experiment, the platelet inhibitor aspirin can reduce inflammation and atherosclerosis in both apolipoprotein E deficient (*apoE*^−/–^) mice and low-density lipoprotein receptor deficient (*Ldlr*^−/–^) mice (45). A previous bioinformatics study proved that the progression of diseases could be affected by Aspirin by acting on drug targeted genes including *PTGS2* (46). However, the specific action of Aspirin in atherosclerotic plaque rupture with thrombosis remains unknown. In the current study, we revealed five hub genes for atherosclerotic plaque rupture with thrombosis including *PTGS2*. Meanwhile, the drug-gene interaction network analysis identified several drug-gene relations including Aspirin-PTGS2. Therefore, we believed that Aspirin may participate in the treatment of atherosclerotic plaque rupture with thrombosis by targeting the *PTGS2* gene.

Our bioinformatic conclusions are firmly anchored in the study's experimental rigor, avoiding over-reliance on in silico analyses. First, all sequencing and bioinformatic inputs derive from a well-validated mouse model, ensuring TDEGs and cell subtypes reflect real pathological progression, not technical noise. Strict scRNA-seq QC further guaranteed data reliability for downstream analyses like UMAP clustering and pseudotime trajectory. Meanwhile, hub genes (COL5A1, VCAN, PTGS2) match literature linking them to plaque stability or thrombosis, and the Aspirin-PTGS2 interaction aligns with clinical anti-thrombotic use. However, future research will be designed to validate the functions of hub gene and fibroblast trajectories among clinical subjects.

## 5 Conclusions

In the present study, fibroblasts might play a vital role in atherosclerotic plaque rupture with thrombosis. In addition, *COL5A1*, *VCAN*, *PTGS2*, *ITGAV* and *ITGA8* might be novel biomarkers for this disease. Moreover, *ITGAV* and *VCAN* might take part in the process atherosclerotic plaque rupture with thrombosis via cell adhesion function. Furthermore, *PTGS2* was target gene for Aspirin in the treatment of atherosclerotic plaque rupture with thrombosis. This investigation provides a new research direction for clinical therapy.

## Data Availability

The raw sequence data reported in this paper have been deposited in the Genome Sequence Archive (Genomics, Proteomics & Bioinformatics 2025) in National Genomics Data Center (Nucleic Acids Res 2025), China National Center for Bioinformation / Beijing Institute of Genomics, Chinese Academy of Sciences (**GSA: CRA034420**) that are publicly accessible at https://ngdc.cncb.ac.cn/gsa.
