# Supplementary material for: Investigation for atherosclerotic plaque rupture with thrombosis in mice based on single-cell sequencing and bioinformatics analysis
Source: Front Cardiovasc Med. 2025 Dec 3;12:1658170. doi: 10.3389/fcvm.2025.1658170 (PMC12709391; doi:10.3389/fcvm.2025.1658170)
Supplement: Supplementary file 1 [file Datasheet1.docx]

Supplementary Information

**Investigation for Atherosclerotic Plaque Rupture with Thrombosis in Mice Based on Single-cell Sequencing and Bioinformatics Analysis**

*Peng Nie^1 #^, Fang Wan^1 #^, Tianbao Yao^1^, Yao Li^2^, Guofeng Yan^2^, Jun Pu^1 *^, Shuxuan Jin^1 *^*

(^#^ Contributed equally)

^1^ Division of Cardiology, Renji Hospital, Shanghai Jiao Tong University School of Medicine, Shanghai, China.

^2^ Department of Laboratory Animal Science, Shanghai Jiao Tong University School of Medicine, Shanghai, China.

*Corresponding authors E-mail: pujun310@hotmail.com; Shuxuan Jin: jinshuxuan111@126.com;

Division of Cardiology, Renji Hospital, Shanghai Jiao Tong University School of Medicine, Shanghai, China, 200025.

**Table 1. The Top 20 nodes in PPI network according to degree**

| **Name** | **Degree** | **Description** |
| --- | --- | --- |
| Il6 | 17 | UP |
| Cd4 | 14 | DOWN |
| Itgav | 12 | UP |
| Pparg | 11 | UP |
| Thbs2 | 11 | UP |
| Col5a1 | 11 | UP |
| Col6a1 | 10 | UP |
| Itga7 | 9 | UP |
| Col6a2 | 9 | UP |
| Fbn1 | 9 | UP |
| Fos | 9 | DOWN |
| Ndufa10 | 9 | UP |
| Acox1 | 8 | UP |
| Pdha1 | 8 | UP |
| Itga9 | 8 | DOWN |
| mt-Nd1 | 8 | UP |
| Acly | 7 | UP |
| Cat | 7 | UP |
| Cyp2e1 | 7 | UP |
| Pcolce | 7 | UP |

**Table 1. The Top 20 nodes in PPI network according to degree.** Notes: PPI, protein-protein interaction network; Degree: the number of interactions associated with certain gene; UP, up-regulation; DOWN, down-regulation.


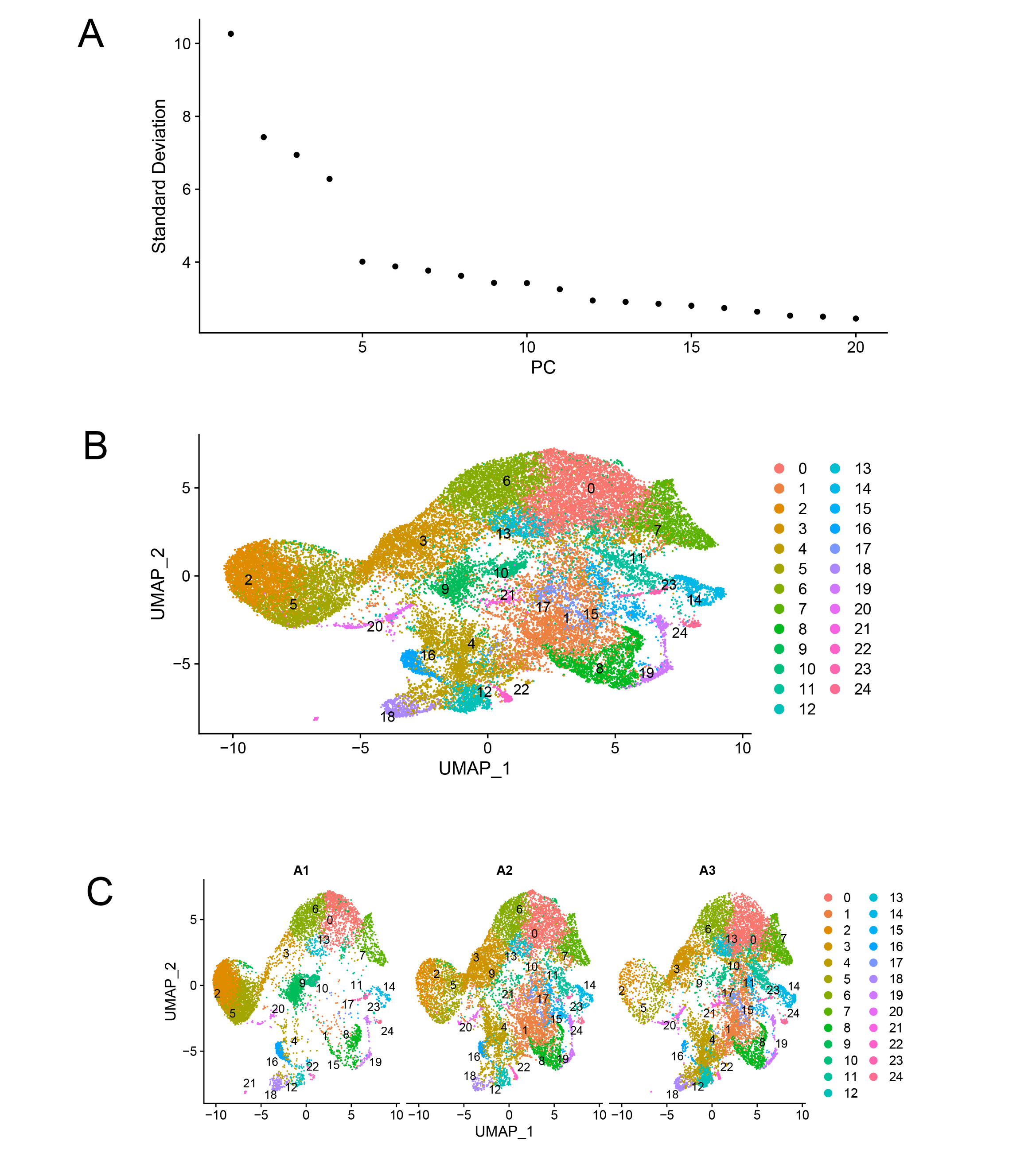


**Supplementary Figure 1. The cell clustering analysis in current study.** A, the result of the linear dimension reduction via Principal Component Analysis (PCA): the Top 10 principal comonents (PCs) contained the dimension with the highest amount of information revealed by elbow plot. B, the Uniform Manifold Approximation and Projection (UMAP) non-linear dimension reduction on all samples: a total of 25 clusters were revealed; different color represented different cluster. C, the UMAP analysis on each group: the different color represented different cluster.


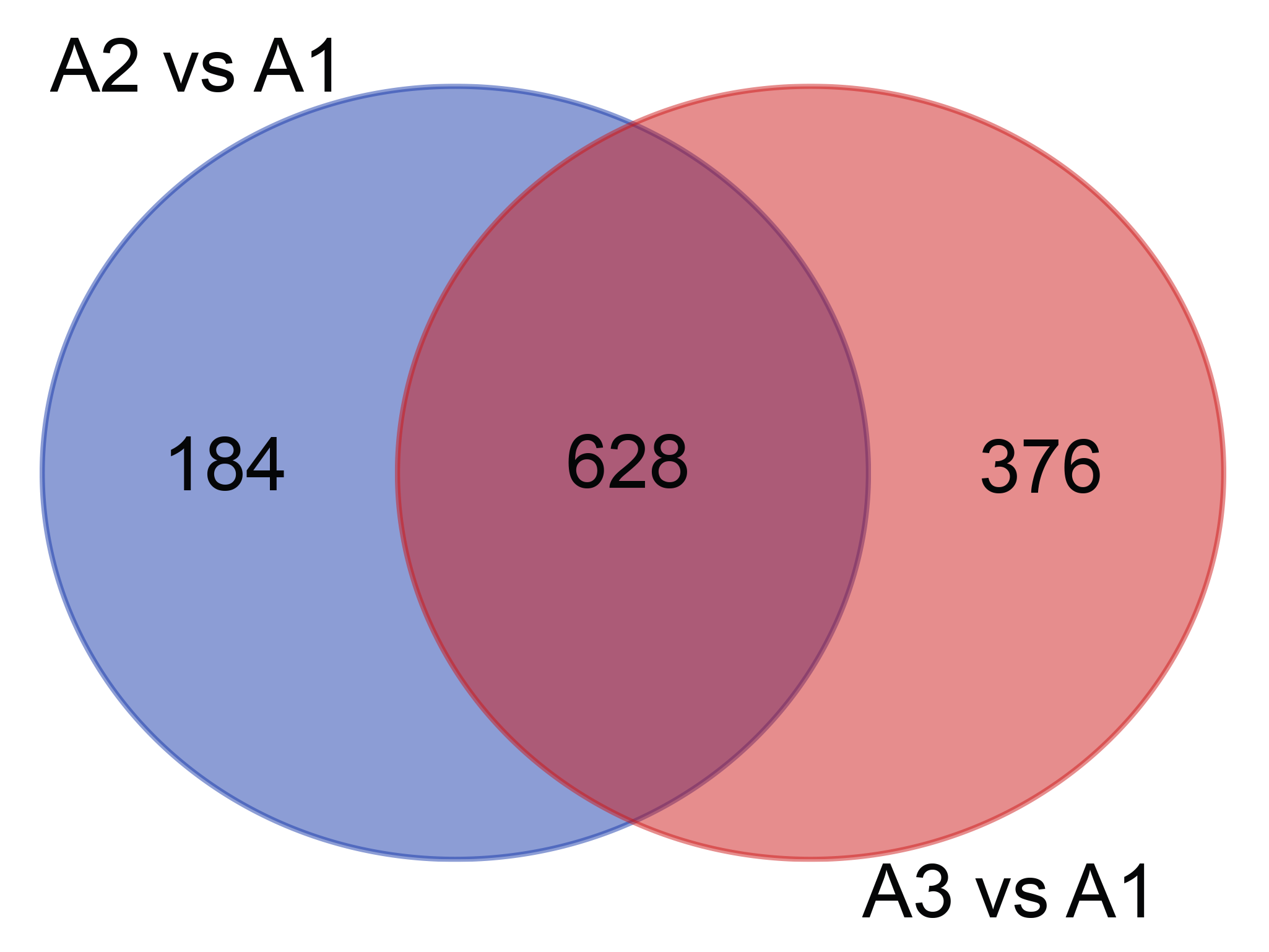


**Supplementary Figure 2. VENN plot revealed 376 T-DEGs based on differentially expressed genes (DEGs) among groups in current analysis.**
